# Fine-tuning alkyl chain length in copper(II) complexes: effects on DNA binding and catalytic cleavage

**DOI:** 10.1007/s10534-026-00840-8

**Published:** 2026-06-18

**Authors:** Maiara I. N. dos Santos, Matheus S. S. Paqui, Giliandro Farias, Edinara Luiz, Ronny R. Ribeiro, Hernan Terenzi, Fernando R. Xavier

**Affiliations:** 1 Chemistry Department, Universidade do Estado de Santa Catarina (Udesc), Joinville, SC Brazil; 2 Chemistry Department, Universidade Federal de Santa Catarina (UFSC), Florianópolis, SC Brazil; 3 Biochemistry Department, CCB, Universidade Federal de Santa Catarina (UFSC), Florianópolis, SC Brazil; 4 Department of Materials Physics and Mechanics, Universidade de São Paulo (USP), São Paulo, SP Brazil; 5 Department of Chemistry, KU Leuven, Celestijnenlaan 200F, 3001 Leuven, Belgium; 6 Chemistry Department, Universidade Federal do Paraná (UFPR), Curitiba, PR Brazil

**Keywords:** DNA catalytic cleavage, Copper(II) complexes, Long alkyl chain ligands

## Abstract

Metal complexes are well known for their DNA cleavage activity. Copper is a metal that stands out for this purpose because it is able to perform cleavage through oxidative mechanisms using the Haber–Weiss cycle. We report two unprecedented copper(II) complexes (**2** and **3**) with long-chain ligands and compare the results with those of a previously reported complex (**1**), which we use as a reference in our studies. They were all properly characterized using a set of physicochemical methods, including elemental analysis, electron paramagnetic resonance, spectroscopic and electrochemical analysis. Density functional theory (DFT) modeling revealed a pseudo-octahedral environment where the ligands are *trans* to each other. The interaction of **1**, **2**, and **3** with salmon sperm DNA was monitored spectrophotometrically, and the binding constants (K_b_) were 4.24 × 10^4^ L mol^−1^, 1.92 × 10^4^ L mol^−1^, and 1.15 × 10^4^ L mol^−1^, respectively. The interactions were supported by docking studies conducted with the CCDC GOLD® docking suite. Finally, all complexes exhibited DNA binding and cleavage properties, but **2** stood out as the most active one (**2**, *k*_obs_ = 0.88 ± 0.09 h^−1^ > **1**, *k*_obs_ = 0.54 ± 0.06 h^−1^ > **3**, *k*_obs_ = 0.50 ± 0.03 h^−1^). These results support the idea that fine-tuning the length of the alkyl chain can enhance DNA-targeting activity and contribute to the development of efficient strategies for catalytic cleavage of nucleic acids.

**Supplementary Information:**

The online version contains supplementary material available at 10.1007/s10534-026-00840-8.

## Introduction

Copper is an essential element for the human body, as it is involved in critical biological processes (Wang et al. 2023). The approximate 80 mg of copper found in the body enables a range of crucial physiological functions, playing vital roles in metalloproteins and a broad spectrum of cellular processes (Choroba et al. 2024; Kakoulidou et al. 2025). Among those processes, the presence of copper in the active sites of various enzymes is particularly notable. For instance, copper-zinc superoxide dismutase (CuZnSOD) plays a fundamental role in cellular defense mechanisms by preventing oxidative damage (Lewandowski et al. 2018). The metal centers of these enzymes catalyze the conversion of superoxide anions into molecular oxygen or hydrogen peroxide, thereby neutralizing endogenously produced free radicals and reactive oxygen species (Lewandowski et al. 2018; Tan et al. 2025). In addition to CuZnSOD, other enzymes that are equally essential to human physiology contain copper in their structure, such as cytochrome c oxidase, which participates in the respiratory process (Berthe et al. 2025), and tyrosinases, which catalyze the initial steps of melanin biosynthesis, the primary pigment in the animal kingdom (Pretzler et al., 2024).

Research involving copper-based compounds encompasses a wide range of therapeutic applications, in which copper contributes to enhanced pharmacological activity. For example, the complexation of pharmaceutical agents with copper has been shown to significantly increase antifungal and antibacterial activity (Dantas et al. 2018; Khaliq et al. 2025; Tabrizi et al. 2025), as well as antiviral (Srivastava et al. 1978; Srivastava 2009), anti-inflammatory (Andrade et al. 2000), anticancer, and catalytic activities (Lištiak et al. 2025). Several compounds have been studied for their ability to promote DNA cleavage (Homrich et al. 2021; Rodríguez et al. 2021; Shaikh et al. 2021). DNA is a significant intracellular target for those compounds that induce cell death by interfering with its replication through genetic damage (Ramesh et al. 2024). The mechanism of DNA cleavage by copper(II) coordination complexes is primarily oxidative and relies on the Cu^II^/Cu^I^ redox cycle. This cycle is typically initiated by a reducing agent such as ascorbate or glutathione, a reaction pathway analogous to the Fenton reaction. This hydroxyl radical then attacks the DNA backbone or guanines, inducing damage (Miao et al. 2018).

Many high-impact copper(II) complexes utilize ligands based on polypyridyl scaffolds, most notably derivatives of 1,10-phenanthroline (phen) or 2,2′-bipyridine (bpy) (Kwon et al. 2014; Li et al. 2013; Wang et al. 2015; Wang et al. 2017). These heterocyclic bases can promote both DNA binding (often via intercalation or groove binding) and proximity, thereby positioning the copper center near the DNA for hydroxyl radical generation and subsequent cleavage. Compounds containing long alkyl chains were evaluated through their amphiphilic behavior towards DNA interaction and cleavage (Shahabadi and Mohammadi, 2012), indicating that the cationic metallic center associated with an alkyl chain can improve DNA binding.

In this context, we selected the bis(2-pyridylmethyl)amine (**bpma**) as a tridentate ligand to be the basis of this work and reference. The secondary amine was replaced with long-chain alkyl groups, and after complexation with copper(II), three complexes were obtained. They were all characterized to obtain valuable structural and physicochemical information, with the main objective of evaluating the influence of the long alkyl chains on the modes and intensities of interaction of these metal complexes with DNA.

## Experimental section

All reactants were used without further purification unless stated otherwise. The reagents used in the DNA cleavage experiments were of molecular biology grade, and all solutions were prepared using Milli-Q ultrapure water. These solutions were sterilized prior to use with 0.22 μm Millex membrane filters (Millipore, USA) to prevent bacterial contamination.

*Elemental analysis* (C, H, and N) determinations were performed on a Perkin-Elmer 2400 CHNS/O Series II analyzer.

*Infrared spectra* were collected on a Perkin Elmer Spectrum 100 device using an ATR module in the range from 4000 to 500 cm^−1^. The data were plotted as transmittance (%) versus wavenumber (cm^−1^).

*NMR spectroscopy* was performed on a Bruker Ascend FT-NMR instrument at 400 MHz using CDCl_3_ as the solvent at 25 °C. Chemical shifts were referenced to tetramethylsilane (TMS, δ = 0.00 ppm).

*GC–MS* spectra were recorded in a Shimadzu QP2010 Plus machine with a 1:50 split and an injector temperature of 250 °C. The temperature programming was from 60 °C (1 min) to 250 °C (5 min), at a heating rate of 10 °C min^−1^. Helium (MS-grade) was used as the carrier gas. The ion source and the MS interface were kept at 250 °C. The mass spectra were recorded between 35 and 600 m*/z*.

The *molar conductivity* for the complexes was evaluated in an MS-Tecnopon mCA150 conductivity meter (previously calibrated with an aqueous 1.00 × 10^–3^ mol L^−1^ KCl solution; Λ_M_ = 146.9 μS cm^−1^) at 25 °C, using 1.00 × 10^–3^ mol L^−1^ spectroscopic grade acetonitrile solutions.

*UV–visible spectra* were obtained at room temperature using a Shimadzu UV3600 Plus spectrophotometer operating from 200 to 1000 nm with quartz cells. Values of molar absorptivity (ε) are given in L mol^−1^ cm^−1^. The spectra were collected under the following conditions: CH_3_CN (100%) and CH_3_CN/HEPES (50% v/v), pH 7.02 (HEPES buffer 0.1 mol L^−1^; ionic strength (NaCl) 0.05 mol L^−1^). The complexes’ concentration was in the order of 10^–3^–10^–5^ mol L^−1^.

The electrochemical behavior of the complexes was investigated in a GAMRY® potentiostat/galvanostat, model 1010E. Cyclic voltammograms were obtained at room temperature in acetonitrile solutions containing 0.1 mol L^−1^ of *n*-Bu_4_NPF_6_ as the supporting electrolyte under an argon atmosphere. The electrochemical cell was composed of three electrodes: platinum (working), platinum wire (auxiliary), and Ag/Ag^+^ (reference). The ferrocene/ferrocenium redox couple Fc/Fc^+^ (E_0_ = 400 mV *vs* NHE) (Gagne et al. 1980) was used as the internal standard. All potentials herein described were referenced to the normal hydrogen electrode (NHE).

Qualitative *electron paramagnetic resonance* spectra of the copper complexes were recorded on a Bruker EMX-microX spectrometer operating at X-band frequency on acetonitrile solutions at 77 K (liquid nitrogen). The spectra were acquired at a microwave power of 0.085 mW and a modulation amplitude of 10 G, and the corresponding spin Hamiltonian simulations were performed using the EasySpin software package (Stoll and Schweiger 2006).

### Theoretical predictions

The geometry of all complexes was optimized in a vacuum with the Orca 5.0.4 software package (Neese 2012) at the DFT level using the PBE0 functional (Perdew et al. 1996, 1997; Zhang and Yang 1998). The basis set chosen was Def2-TZVP for the copper atom, Def2-TZVP(-f) for the oxygen and nitrogen atoms, and Def2-SVP for the other atoms (Schäfer et al. 1994; Weigend and Ahlrichs 2005). Dispersion effects were included using Grimme’s D3 correction with Becke-Johnson (BJ) damping (Grimme et al. 2010, 2011). The evaluation of four-center integrals was accelerated using the RIJCOSX algorithm, which employs the resolution-of-identity approximation for the Coulomb part (RIJ) and the chain-of-spheres approach for the Fock exchange (COSX) (Kossmann and Neese 2010; Zhang and Yang 1998). The RIJ requires the specification of an auxiliary basis set for the Coulomb part (Def2/J) and a numerical integration grid for the exchange part (DefGrid-2) (Zhang and Yang 1998). Analytical harmonic vibrational frequency calculations were conducted to verify if the ground state is a minimum on the potential energy surface. To simulate the absorption spectra, the time-dependent density functional theory under the Tamm-Dancoff approximation (TD-DFT/TDA) (Perdew et al. 1996) was employed to obtain the first 50 excitations, using the same calculation protocol, including the solvent correction, using the linear response conductor-like polarizable continuum model (LR-CPCM) method (Perdew et al. 1997). The 3D representations of the complexes were obtained using Chemcraft (www.chemcraftprog.com).

### DNA assays: UV–Vis and circular dichroism

DNA binding experiments were performed with salmon sperm DNA (ssDNA) with a concentration around 10^–5^ mol L^−1^ in a 2.00 × 10^–2^ mol L^−1^ HEPES buffer (pH 7.00), Ionic strength (*I*) was kept at 2.00 × 10^–2^ mol L^−1^ with NaCl, and CH_3_CN solutions of the complexes **1**, **2** and **3** with a final ratio of 50% (v/v) Buffer/CH_3_CN. The final concentration of the ssDNA solution was determined from the absorbance at 260 nm (ε = 6600 L mol^−1^ cm^−1^) (Mohamadi et al. 2015). The complexes were titrated with different ssDNA concentrations, and the spectra were recorded. The absorbance values were corrected for dilution, and the intrinsic binding constants K_b_ were obtained by fitting the values to the Benesi-Hildebrand equation as follows (Mohamadi et al. 2015):

$$\frac{A0}{(A-A0)}=\frac{\varepsilon f}{(\varepsilon b-\varepsilon f)}+\frac{\varepsilon f}{(\varepsilon b-\varepsilon f)}\frac{1}{Kb\left[DNA\right]}$$

where A_0_ is the initial complex absorbance, A is the complex absorbance in the presence of DNA, ε_f_ and ε_b_ are the absorptivity of the free and bound complexes, respectively. Based on the plot of A_0_/(A – A_0_) *vs* 1/[DNA], the intercept-to-slope ratio gives the value of K_b_ (Mohamadi et al. 2015).

Circular dichroism spectra were recorded using a JASCO J-815 CD spectropolarimeter (Jasco, USA). The experiments were conducted in a 2-mm optical path quartz cuvette containing 40 μL of HEPES buffer (10 mmol L^−1^), 80 μL of CT-DNA (type XV, Sigma) stock solution (200 μmol L^−1^), and 280 μL of water. CT-DNA was titrated with 2 mmol × L^−1^ solutions of the compounds with [complex]/[DNA] ratios ranging from 0.05 to 2.5. CD spectra were recorded in triplicate, with each scan representing the accumulation of three individual spectra. Control experiments, including a blank and a complex without DNA, were also performed**.**

### DNA assays: cleavage studies

Plasmid DNA, pBlue Script II SK (pBSK-II, 2961 bp, Stratagene), was used as a substrate. The plasmid was amplified by transformation into competent DH5-α *Escherichia coli* cells, as previously described (Ausubel et al. 1999), and purified using the manufacturer’s protocol (PureYield™ Plasmid Maxiprep System). The DNA concentration was determined by UV–Vis spectroscopy at 260 nm (ɛ = 13,200 L mol^−1^ cm^−1^), and its integrity was confirmed by agarose gel electrophoresis. For the DNA cleavage assays, reactions were prepared in a 100 µL microcentrifuge tube as follows: 330 ng of plasmid DNA (2 µL, 25 mmol L^−1^ bp), 2 µL of buffer (100 mmol L^−1^), and 5 µL of compounds **1**, **2** and **3** (various concentrations, CH_3_CN as the solvent), with the volume adjusted to 20 µL using water. When explicated, 2 µL of sodium L-ascorbate (500 µmol L^−1^) was also included. For pH effect studies, the buffers were 2-(*N*-morpholino)ethanesulfonic acid (MES, pH 6.0), 4-(2-hydroxyethyl)piperazine-1-ethane-sulfonic acid (HEPES, pH 7.0 and pH 8.0), and 2-(*N*-cyclohexylamino)ethanesulfonic acid (CHES, pH 10.0). To assess the effect of ionic strength, 2 µL of NaCl (500 mmol L-1) was incubated with DNA for 15 min before the compounds were added. For a groove interaction study, 2 µL of 4′,6-diamidino-2-phenylindole (DAPI, 500 µmol L^−1^) and methyl green (MG, 500 µmol L^−1^) were incubated with DNA for 30 min before adding the compounds. The reactive oxygen species (ROS) assay was performed using dimethyl sulfoxide (DMSO), D-mannitol, L-histidine, and superoxide dismutase 1 (SOD) (4 µL, 200 µmol L^−1^). All experiments included a control group to account for spontaneous DNA degradation under the experimental conditions. The final concentration of each species is detailed in the figure’s captions. At the end of each experiment, reactions were quenched by adding 5 µL of loading buffer solution, containing 50 mmol L^−1^ of Tris(hydroxymethyl)aminomethane hydrochloride (TRIS, pH 7.5); 0.01% of bromophenol blue (pH 8.0), 50% glycerol, and 250 mmol L^−1^ ethylenediaminetetraacetic acid (EDTA). The samples were analyzed by gel electrophoresis on 1% agarose gel containing 0.3 μg mL^−1^ of ethidium bromide, run in 0.5 × TRIS–borate-EDTA buffer (TBE) (44.5 mmol L^−1^ of TRIS, pH 8.0; 44.5 mmol L^−1^ of boric acid, 1.0 mmol L^−1^ of EDTA) for 100 min at 90 V. The gels were documented using a DigiDoc-It system (UVP, USA), and the relative proportions of plasmid DNA topoisomers were quantified by densitometry using the Image Processing and Analysis in Java (ImageJ) software package.

### In silico* studies*

In silico analysis (binding mode) for complexes **1**, **2**, and 3 with double-stranded DNA was performed using the Genetic Optimization for Ligand Docking – GOLD 2025.1.0 software program (Cambridge Crystallographic Data Centre, Cambridge, CB2 1EZ, UK) (Jones et al. 1997). Calculated structural data from DFT studies for all complexes were employed, and the DNA dodecamer d(GCGCAATTGCGC)_2_ was retrieved from the Protein Data Bank (PDB ID: 2BNA) (Drew et al. 1982). This DNA dodecamer adopts the B-conformation, the most common inside the cell and, thus, the most studied. Crystallization water molecules from the DNA dodecamer were removed, polar hydrogen atoms were added to the DNA following tautomeric states, and ionization data were inferred by the GOLD 2025.1.0 software package at pH 7.4, exploring a 5.0 Å radius around the two main possible binding sites: major and minor grooves. The GoldScore standard function was used, once optimized for predicting ligand binding positions, and considers factors such as H-bonding energy, van der Waals energy, metal interactions, and ligand torsion strain [1]. The DNA-complex adducts were visualized and further analyzed with ChimeraX v.1.4 (Goddard et al. 2018; Pettersen et al. 2021).

### Synthesis

#### Synthesis of ligands

Bis-(2-pyridylmethyl)amine (**bpma**), 6-(bis(pyridyl-2-methyl)amino)hexan-1-ol (**L**^**C6OH**^), and 10-(bis(pyridyl-2-methyl)amino)decan-1-ol (**L**^**C10OH**^) were synthesized as previously described (Melotti et al. 2021; Schattschneider et al. 2019). The complete synthesis path is provided in the Supporting Material.

#### Synthesis of [Cu(bpma)_2_](ClO_4_)_2_ · i-PrOH (1)

Complex **1** was prepared at a stoichiometric ligand/metal ratio of 2:1, following the previous reported procedure (Huang et al. 2000; Palaniandavar et al. 1996). A solution of 0.5 mmol of Cu(ClO_4_)_2_ · 6 H_2_O in 5 mL of methanol was added dropwise to a stirring methanolic solution (10 mL) of **bpma** (1.0 mmol) at 40 °C. The initial brown solution changed to dark blue. After the complete addition of copper perchlorate, the solution was stirred at room temperature for 30 min, then left to crystallize at room temperature. After three days, a dark blue precipitate was obtained and washed with cold isopropyl alcohol and ethyl ether. The solid complex was dried under reduced pressure. The yield was 80%. MW: 721.05 g mol^−1^. C_27_H_34_Cl_2_CuN_6_O_9_. Calculated: C 44.98; H 4.75; N 11.66. Experimental: 44.61; H 4.93; N 11.49. (corresponding to [Cu(bpma)_2_](ClO_4_)_2_ · C_3_H_8_O)_._ IR (ATR, cm^−1^):* ʋ* (N–H) 3240; *ʋ* (C = C; C = N) 1609–1444;* ʋ* (Cl–O) 1050; *δ* (C–H_ar_) 768–620. Molar conductivity (CH_3_CN, 1.0 × 10^–3^ mol L^−1^) Λ_M_ = 293 μS cm^−1^ (2:1 electrolyte).

#### Synthesis of [Cu(L^C6OH^)_2_](ClO_4_)_2_ · i-PrOH · H_2_O (2)

Complex **2** was prepared at a stoichiometric ligand/metal ratio of 2:1. A solution of 0.5 mmol of Cu(ClO_4_)_2_ · 6 H_2_O in 5 mL of methanol was added dropwise to a stirring methanolic solution (10 mL) of **L**^**C6OH**^ (1.0 mmol) at 40 °C. The initial brown solution changed to dark green. After the addition of copper perchlorate was complete, the solution was stirred at room temperature for 30 min. The solvent was evaporated under reduced pressure, and the complex was washed with cold isopropyl alcohol and ethyl ether. The residual solvents were removed under reduced pressure, yielding a dark green oil. The yield was 60%. MW: 939.39 g × mol^−1^. C_39_H_60_Cl_2_CuN_6_O_12_. Calculated: C 49.87; H 6.44; N 8.95. Experimental: 50.14; H 6.67; N 8.62. (corresponding to [Cu(L^C6OH^)_2_](ClO_4_)_2_ · *i-PrOH* · H_2_O)_._ IR (ATR, cm^−1^): *ʋ* (O–H) 3523; *ʋ* (C = C; C = N) 1611–1447;* ʋ* (Cl–O) 1075; *δ* (C–H_ar_) 764–621. Molar conductivity (CH_3_CN, 1.0 × 10^–3^ mol L^−1^) Λ_M_ = 297 μS cm^−1^ (2:1 electrolyte).

#### Synthesis of [Cu(L^C10OH^)_2_](ClO_4_)_2_ · 3H_2_O (3)

Complex **3** was prepared at a stoichiometric ligand/metal ratio of 2:1. A solution of 0.5 mmol of Cu(ClO_4_)_2_ · 6H_2_O in 5 mL of methanol was added dropwise to a stirring methanolic solution (10 mL) of **L**^**C10OH**^ (1.0 mmol) at 40 °C. The initial brown solution changed to dark blue. After the addition of copper perchlorate was complete, the solution was stirred at room temperature for 30 min. The solvent was evaporated under reduced pressure, and the complex was washed with cold isopropyl alcohol and ethyl ether. The residual solvents were removed under reduced pressure, yielding a dark blue oil. The yield was 60%. MW: 1027.54 g mol^−1^ C_44_H_72_Cl_2_CuN_6_O_13_. Calculated: C 51.43; H 7.06; N 8.18. Experimental: 51.21; H 6.67; N 8.62. (corresponding to [Cu(L^C10OH^)_2_](ClO_4_)_2_ · 3H_2_O). IR (ATR, cm^−1^): *ʋ* (O–H) 3525; *ʋ* (C = C;C = N) 1608–1445;* ʋ* (Cl-O) 1078; *δ* (C–H_ar_) 759–621. Molar conductivity (CH_3_CN, 1.0 × 10^–3^ mol L^−1^) Λ_M_ = 192 μS cm^−1^ (2:1 electrolyte).

## Results and discussion

### Ligand synthesis and characterization

Ligands were synthesized according to the literature (Melotti et al. 2021; Schattschneider et al. 2019). The main differences between **bpma**, **L**^**C6OH**^**,** and **L**^**C10OH**^ are the presence and the length of the aliphatic chain, which acts as an uncoordinated group. While **bpma** consists of bis-(2-pyridylmethyl)amine,** L**^**C6OH**^ and **L**^**C10OH**^ also contain a 6- or a 10-carbon chain and a terminal alcohol. These modifications maintain the same coordination sphere for the desired complexes while allowing us to explore the effects of these side chains on their physicochemical properties and biological activities.

All the relevant characterization and discussion regarding the described ligands are provided in the supporting material (Figures S1–S7). Infrared spectroscopy and nuclear magnetic resonance (^1^H and ^13^C NMR) data are in strong agreement with previously reported results for these ligands (Melotti et al. 2021; Schattschneider et al. 2019).

### Complexes’ characterization

**Fig. 1:**
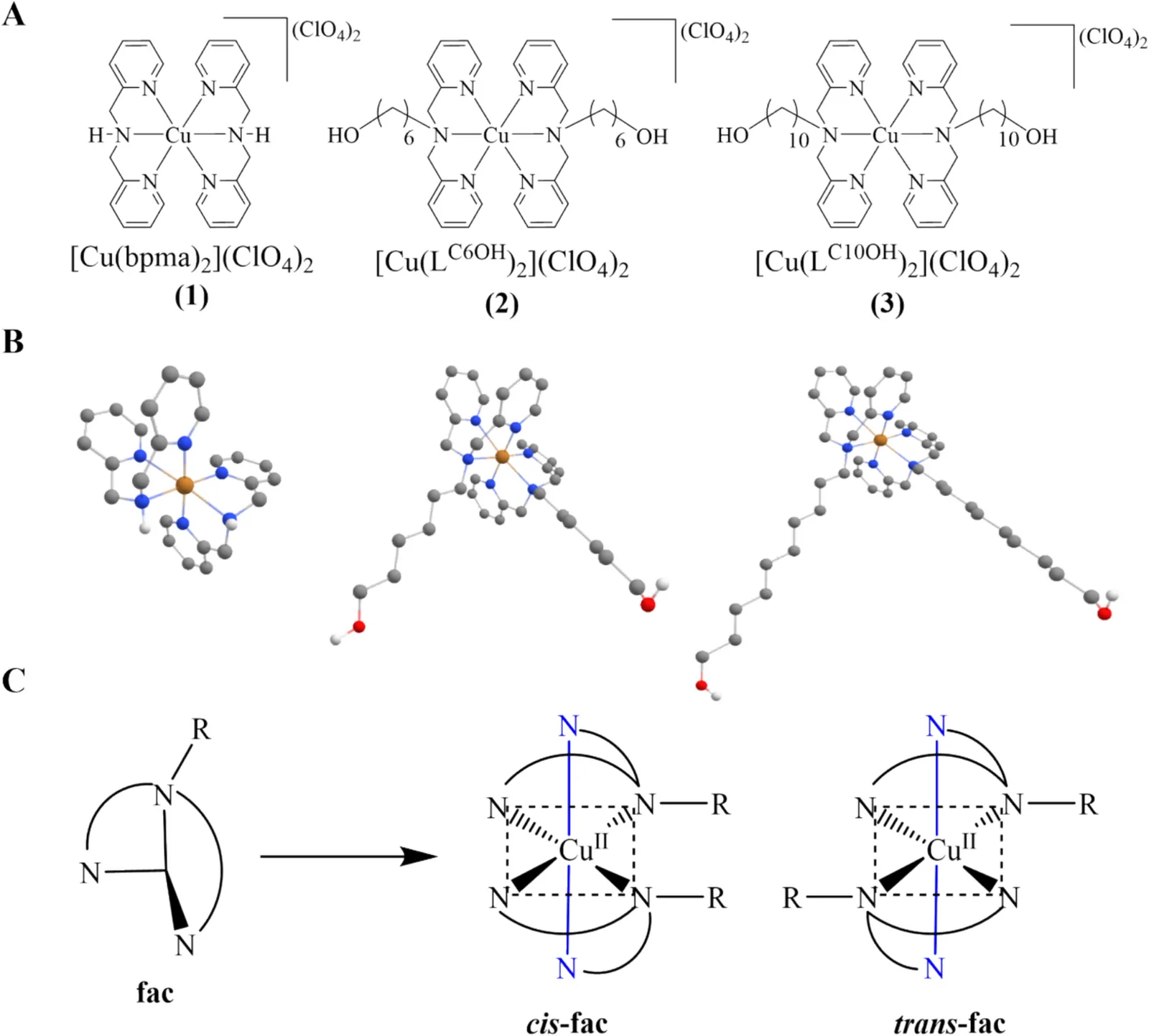
**A** Proposed structures of complexes **1**, **2**, and **3** synthesized and investigated in this study. **B** Molecular arrangement *cis-fac* for **1**–**3** obtained at DFT level of theory. Atoms: Cu (brown); nitrogen (blue); carbon (grey); oxygen (red); hydrogen (white), or were omitted for clarity. **C** Possibilities of molecular arrangement of the donor atoms surrounding the copper in a facial structure. (Color figure online)

Based on **bpma**, **L**^**C6OH**^**,** and **L**^**C10OH**^, symmetric mononuclear copper(II) complexes were synthesized (Fig. 1A) to evaluate their effect on DNA interaction and cleavage. While complex **1** forms a more compact structure (Huang et al. 2000; Palaniandavar et al. 1996), in which the organic portion is constrained around the metal center, complexes** 2** and **3** incorporate alkyl side chains of six and ten carbon atoms, respectively, increasing their molecular size. These differences directly impact their physicochemical properties and, more importantly, influence their mode of interaction with DNA, ultimately affecting their biological activity.

### Solid state and DFT structural analysis

Elemental analysis of complexes **1**, **2**, and **3** is consistent with the proposed chemical formula, given the presence of hydration molecules (see the complex synthesis for details). Infrared spectroscopy for complexes** 1**,** 2**, and** 3** agrees with the reported bands of each ligand (Figure S8) (Anderegg et al. 1975; Huang et al. 2000; Melotti et al. 2021). All relevant ligand bands, such as C–N, C–C, and C–H, appear in the complex spectra with slight shifts due to coordination. In addition, a broad band at ~ 1070 cm^−1^ indicates the presence of perchlorate as a counterion (Table S2).

The vibrational frequencies were calculated for all three complexes, showing a good correlation between theoretical and experimental results. Perchlorate counterions were not considered in the modeling; therefore, the corresponding band is absent from the calculated spectra.

DFT calculations were performed to provide further insight into the likely structures of complexes** 1**,** 2**, and** 3**. First, we considered that the tridentate ligand would coordinate in a *facial* (*fac*) arrangement due to the typical increase in bond length along the* z*-axis, thereby increasing the energy required for a *meridional* (*mer*) arrangement. In the *fac*-arrangement, coordination can occur in either *cis* or *trans* spatial configuration. Calculations indicate that the *cis-fac* arrangement for complex** 1** is approximately 9.75 kJ mol^−1^ more stable than the *trans-fac* conformation (Fig. 1C). Therefore, at room temperature, 98% of the species would adopt the *cis-fac* configuration. For complexes **2** and **3**, this energy difference is even larger, indicating that isomerism is negligible for these compounds, which can only assume a *cis-fac* conformation. The molecular arrangements are shown in Fig. 1B.

For all complexes, the coordination environment is pseudo-octahedral, with Cu(II) coordinated to six nitrogen atoms: two from amines and four from pyridine (*py*) groups. In these complexes, each amine is *trans* to a pyridine group from the second ligand, while the remaining pyridine pairs are *trans* to each other (Fig. 1B). The average Cu–N bond lengths for complexes **1**, **2**, and** 3** are 2.175 Å, 2.206 Å, and 2.215 Å, respectively, which are in good agreement with literature values (Huang et al. 2000; Palaniandavar et al. 1996). The increase in bond length for complexes** 2** and **3** is due to the presence of the alkyl chain. The axial bonds in each complex are longer than the equatorial ones, because of the Jahn–Teller effect. The progressive increase in axial bond length from complex** 1** to complex** 3** indicates that the size of the alkyl chain influences the coordination sphere. For complex** 1**, the longest bond is 2.482 Å (py_1_(a)), and the shortest is 2.026 Å. In Complexes** 2** and** 3**, these values are 2.496 Å and 2.016 Å, and 2.513 Å and 2.012 Å, respectively (Scheme S1 and Table S3).

### Solution studies

Conductometric measurements suggested a 2:1 ratio of anions to cations for complexes** 1**, **2**, and** 3**, indicating a cationic complex with two units of perchlorate as counterions. The results in CH_3_CN showed conductance values of 293 µS cm⁻^1^ and 297 µS cm^−1^ for complexes** 1** and** 3**, respectively, which are in good agreement with the literature. For complex** 2**, this value was slightly lower than expected (192 µS cm^−1^). However, according to the literature, it is still more consistent with a 2:1 electrolyte ratio than with a 1:1 (Geary 1971).

**Fig. 2:**
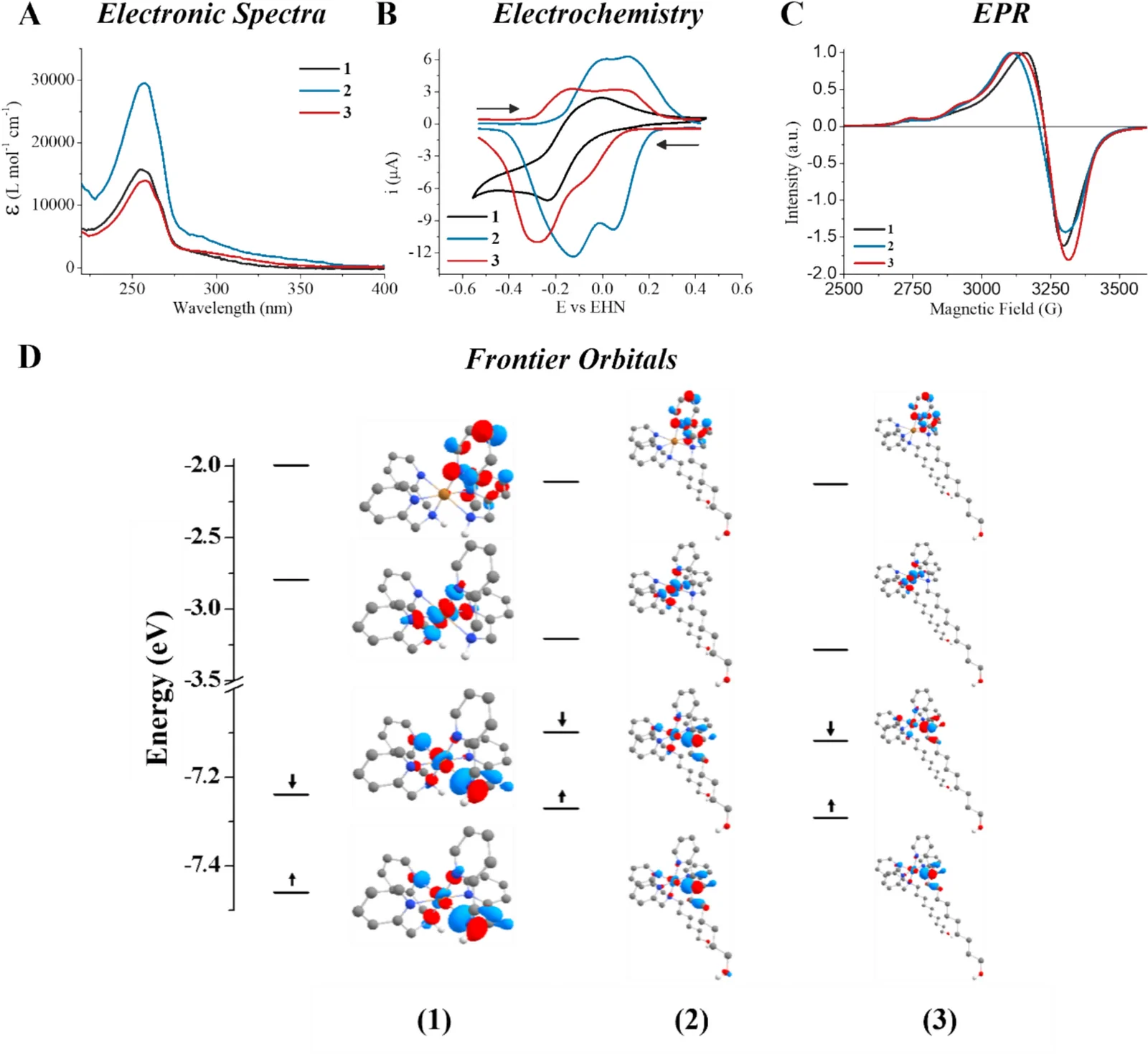
**A** Electronic spectra of **1**, **2** and **3** in CH_3_CN. Conditions: [**1** and **2**] = 2.5 × 10^–5^ mol L^−1^ and 1.0 × 10^–3^ mol L^−1^ [**3**] = 5.0 × 10^–5^ mol L^−1^. **B** Electronic paramagnetic resonance spectrum and parameters of **1**,** 2**, and **3** in CH_3_CN at 77 K. C Voltammograms of **1**, **2**, and **3**. Conditions [C] = 1.0 × 10^–3^ mol L^−1^; [tetrabutylammonium hexafluorophospate] = 0.1 mol L^−1^. Electrodes: working—glassy carbon; auxiliary—platinum wire; reference—Ag/Ag^+^. (**1**) Cyclic voltammetry: scan rate 25 mV s^−1^; (**2** and **3**) square wave voltammetry: pulse: 25 mV; frequency: 25 Hz; scan step: 2.0 mV. **D** Frontier orbitals for **1** (left), **2** (middle), and **3** (right)

The absorption spectra of all compounds were recorded in CH_3_CN (Fig. 2DA, 200–400 nm for **1**–**3**; Figure S9, Ligand-Complex comparison; Figure S10, 400—1000 nm for complexes **1**–**3**. See Table S4 for a summary of absorptions bands). The broadening of the bands in this region is related to the Jahn–Teller distortion, which is common for octahedral Cu(II) centers, and the maximum absorbance is consistent with similar results reported in the literature (Hartman et al. 2003; Huang et al. 2000). The λ_max_ also helps explain the complex colors, with values of 634 nm, 676 nm, and 610 nm for complexes** 1**,** 2**, and** 3**, respectively. For complexes** 1** and** 3**, this absorption occurs in the red-to-orange region, producing a green color in the visible spectrum for each complex. However, for complex** 2**, this absorption occurs at a longer wavelength, giving rise to a blue color.

At higher energies, all complexes show an intense band around 258 nm. Time-dependent DFT (TD-DFT) calculations indicates that, for complex** 1**, this band corresponds to a charge-transfer (CT) transition from the *d*-electrons of Cu(II) to the π* orbitals of pyridine, along with ligand-based transitions, such as *π* → π* from pyridine or *n* → π* from the secondary amine (see Fig. 2D and Table S5). For complexes** 2** and** 3**, these transitions are similar to those described above, but the CT transition contributes less. Additionally, the entire theoretical absorption spectra were simulated for all compounds and showed good agreement with the experimental ones (Figure S10). Finally, the stability of complexes** 1**,** 2**, and** 3** in solution (50% v/v CH3CN/HEPES buffer) was monitored spectroscopically from 220 to 350 nm over 2 h (Figure S11). For all complexes, no significant spectral changes were observed over time, suggesting stability under such conditions.

Electrochemical properties were also evaluated in CH_3_CN (Fig. 2B). All redox potentials are referenced to NHE, using the ferrocene/ferrocenium pair (E_1/2_ = 0.400 V *vs* NHE) as an internal standard. For complex** 1**, the electrochemical potential was measured by cyclic voltammetry, and the process at −0.12 V *vs* NHE is attributed to the redox pair Cu(II) + e^−^ ⇌ Cu(I). For complexes** 2** and **3**, due to the proximity of the redox processes, square-wave voltammetry was used to determine the redox parameters (Table S4). Two processes were observed in these cases. The Cu(II) reduction/oxidation occurs at −0.06 V *vs* NHE for complex** 2** and at 0.19 V *vs* NHE for complex **3**. The other process, at 0.07 V *vs* NHE (the same for both complexes** 2** and** 3**), is likely associated with the electrochemical generation of a Cu(I) species in solution, resulting in the second process (Bard, 2001; Gagne et al. 1980; Hartman et al. 2003; Karlin, 1984; Palaniandavar et al. 1996).

Electron paramagnetic resonance was measured in a frozen CH_3_CN solution at 77 K. The spectra for **1**, **2**, and **3** (Fig. 2C) show three spectroscopically equivalent complexes, consistent with Cu(II) metal centers. For theoretical comparisons, see Figure S12. All exhibit axial arrangements, as confirmed by the *g*-tensor values (*g*_z_ > *g*_x_ = *g*_y_), suggesting a *dx*^*2*^*-y*^*2*^ ground state with shortened equatorial axes. These features are typical for Cu(II) complexes with Jahn–Teller distortion, and they are in strong agreement with the DFT calculations and spectrophotometric evidence discussed above (Garriba & Micera 2006; Ju et al. 2024). Furthermore, the relation between the *A*z and *g*_z_ components supports a four-nitrogen coordination at the Cu(II) equatorial plane (Peisach & Blumberg 1974).

### DNA assays

#### UV–Vis and CD titrations

To assess whether complexes **1**, **2**, and **3** interact with nucleic acids, they were analyzed using salmon sperm DNA (ssDNA) by spectrophotometric techniques. In this method, the intrinsic binding constant (K_b_) is used to evaluate the stability of the complex/DNA adduct and is determined using the Benesi-Hildebrand equation.

**Fig. 3:**
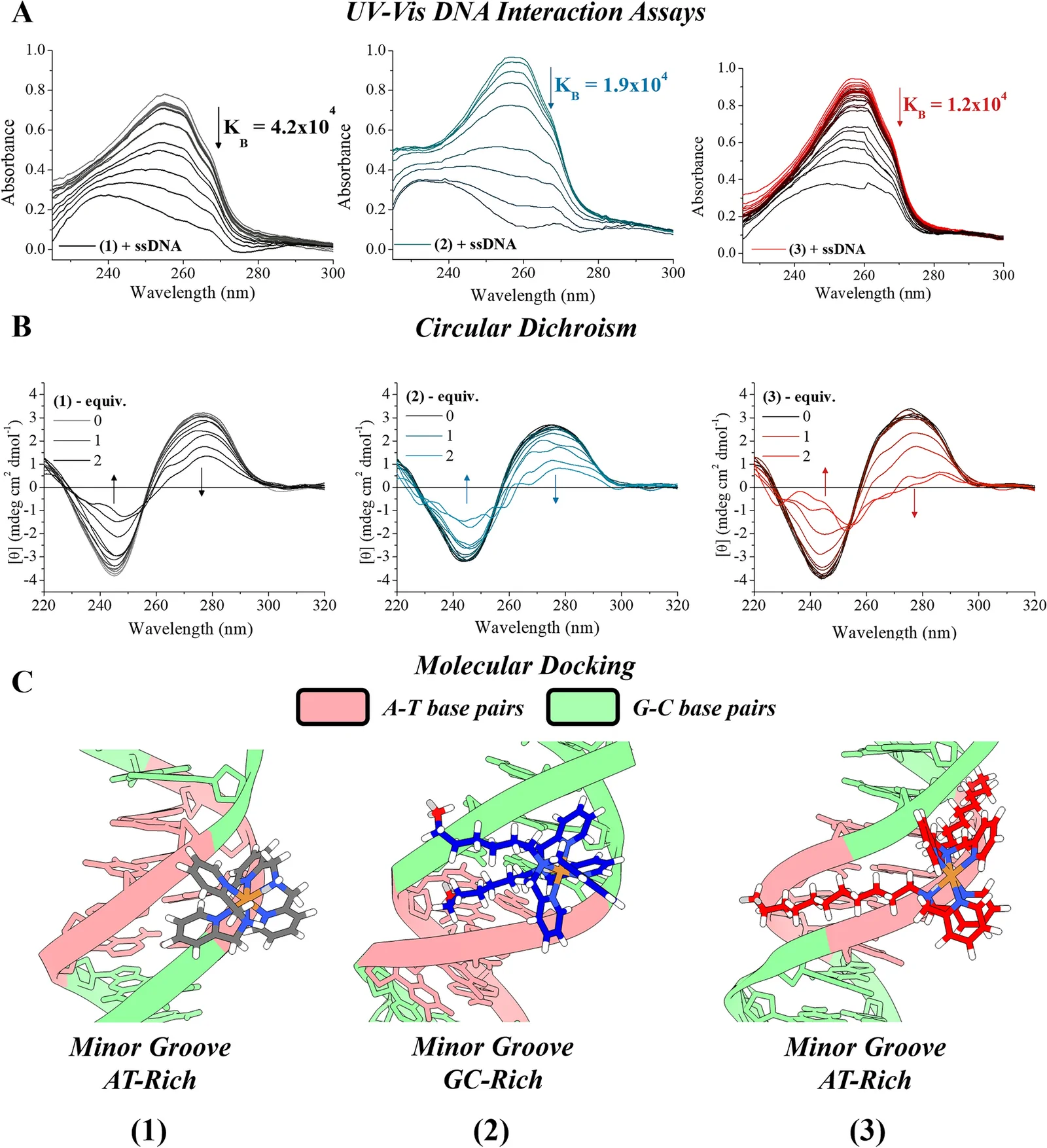
**A** UV–Vis DNA interaction assay using ssDNA as substrate for complexes **1**,** 2** and** 3**. [ssDNA] = 2.00 × 10^–3^ mol L^−1^ was titrated over complexes in CH_3_CN/HEPES (pH 7.0) (50% v/v); [**1**] = 1.5 × 10^–4^ mol L^−1^; [**2**] = 5.0 × 10^–4^ mol L^−1^; e [**3**] = 1.0 × 10^–4^ mol L^−1^**. B** Circular dichroism spectra of DNA **1**,** 2**, and **3.** DNA = 200 µmol L^−1^, [**1**,** 2** and **3**] = 10 to 400 µmol L^−1^, [Buffer] = HEPES (10 mmol L^−1^, pH 7.0), T = 25 °C. **C** Close-up molecular docking structures highlighting minor groove interactions for compounds **1**,** 2**, and **3**

All studied complexes showed significant hypochromism between 230 and 280 nm, i.e., the absorption decreased as DNA was added. For complex **1**, a 77% decrease in absorbance at 254 nm was observed (Fig. 3A). For complexes **2** and **3**, absorption reductions of 88% and 63%, respectively, were observed. This behavior suggests that the interaction primarily occurs through the DNA grooves and/or via electrostatic interactions (Melotti et al. 2021; Schattschneider et al. 2019).

To assess the magnitude of the interaction between the metal complexes and DNA, the intrinsic binding constants (K_b_) were determined using the Benesi-Hildebrand method, with K_b_ obtained from the ratio of the intercept to the slope of the linearization (Figure S13C). The K_b_ values calculated for complexes **1**, **2**, and **3** were 4.2 × 10^4^ L mol^−1^, 1.9 × 10^4^ L mol^−1^, and 1.2 × 10^4^ L mol^−1^, respectively, indicating a similar binding strength between the three compounds. Cobalt(III) compounds containing long alkyl chains resulted in similar K_b_ with DNA (Kumar et al. 2009). The calculated K_b_ for **bpma** was 5.9 × 10^3^ L mol^−1^ and, for **L**^**C6OH**^, 5.3 × 10^3^ L mol^−1^ (Figure S13, A and B). The K_b_ for the ligand **L**^**C10OH**^ measured by Melotti and Coworkers (2021) was 5.2 × 10^3^ L mol^−1^, in good agreement with our data.

Considering the K_b_ data for both the free ligands and the metal complexes, an increase in DNA-binding constants is observed upon metal ion incorporation. 50% acetonitrile was used in UV–Vis experiments to guarantee that no precipitation of the alkyl chain compounds would occur during the titration experiments.

CD experiments were performed with CT-DNA to investigate the interaction modes of the compounds with the nucleic acid structure (Fig. 3B). The titration of the complexes on DNA led to changes in the characteristic B-DNA bands at 245 nm (right-handed helicity) and 275 nm (base stacking) (Ausubel et al. 1999; Dimitrakopoulou et al. 2008). The displacement of both bands upon titration of the complexes indicates the loss of the DNA’s right-handed helical structure. These alterations imply that the compounds can bind to the DNA grooves and interact via electrostatic forces. Since there was no increase in the 275 nm band, no intercalative binding mode was observed (Dhar et al. 2005; Gabriel et al. 2022). Compounds **2** and **3** appear to induce a greater displacement of the DNA’s right-handed helicity compared to **1**. These results were expected, as the alkyl chains are likely to interact with the DNA grooves, as observed in our previous work (Melotti et al. 2021).

### Molecular docking

We divided the dodecamer (PDB 2BNA) into four regions, which were individually evaluated for docking. These four regions are: the AT-rich minor groove, the AT-rich major groove, the GC-rich minor groove, and the GC-rich major groove. The data for the strongest interactions in each region for **1**, **2**, and** 3** are presented in Table S6, and the best-scoring structures for each compound are in Fig. 3C.

**Fig. 4:**
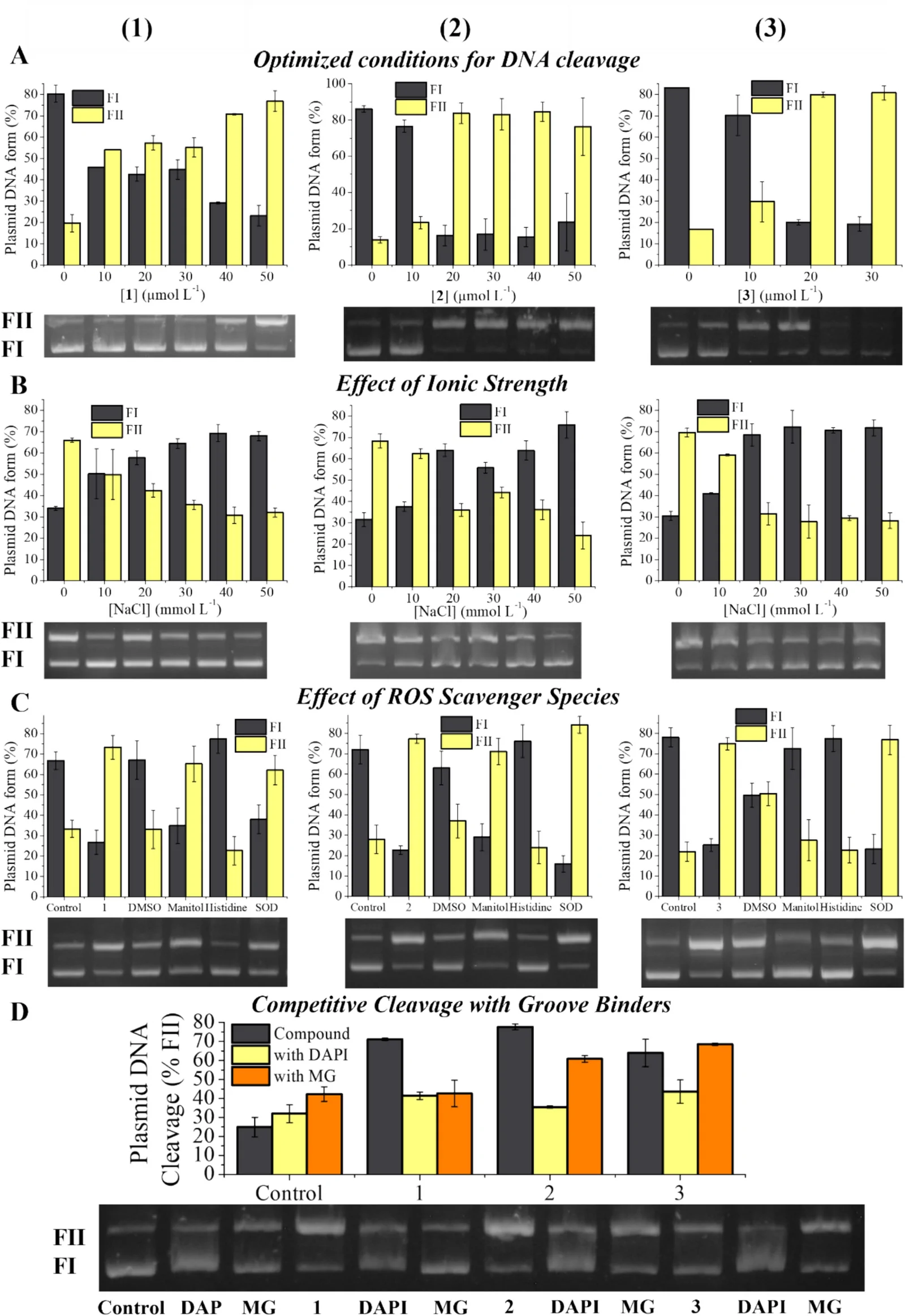
**A** Comparison of DNA cleavage by compounds **1**,** 2**, and **3**. DNA = 330 ng (25 µmol L^−1^), [**1**,** 2**, and** 3**] = 10–50 µmol L^−1^, [Sodium L-ascorbate] = 50 µmol L^−1^, [Buffer] = HEPES (10 mmol L^−1^, pH 8.0), T = 37 °C, t = 2 h. **B** Effect of ionic strength on supercoiled DNA cleavage by **1**, **2**, and **3**. [**1**,** 2**, and** 3**] 20 µmol L^−1^, [NaCl] = 0–50 mmol L^−1^. **C** Effect of ROS scavenger species on DNA cleavage by **1**,** 2**, and** 3**. [**1**,** 2**, and** 3**] 20 µmol L^−1^, [Scavengers] = 50 µmol L^−1^. **D** DNA groove interaction studies. [**1**,** 2** and **3**] 20 µmol L^−1^, [DAPI] = [MG] = 50 µmol L^−1^. Conditions not mentioned are the same as **A**’s

Most interactions for **(1)** occur on the minor groove (GoldScore (GS) 43.43 and 37.55 for minor groove AT and GC-Rich regions, respectively), even when interactions are constrained to the specific regions using a 5 Å sphere for docking calculations. Furthermore, the lack of significant differences between AT or GC-rich regions suggests that the DNA interaction would happen primarily on the minor groove, but weaker in comparison with other compounds, where selectivity is pronounced, allowing for promiscuity of the binding site for cooperative binding at multiple DNA sites, as suggested by the competitive binding assay with groove binders (Fig. 4D). The interactions are strictly driven by van der Waals forces for **(1)**, meaning that the secondary amine protons from **bpma** do not help increase the compound interaction and, therefore, can be replaced by an alkyl chain without a significant impact. The best structures for each case are shown in Figure S14.

For **(2)**, we executed the docking calculations the same way (Table S6). The GoldScore data suggest similar interactions across all regions of the dodecamer; however, the minor groove (GS 47.24 and 53.41 for AT and GC, respectively) has a considerably better fit than the major groove (GS 40.47 and 40.14 for AT and GC, respectively). There are no substantial differences in the AT/GC comparison, indicating that the interaction is primarily driven by the size of the hydrophobic pockets, in a similar, but stronger, way than **(1)**. Hydrogen bonds become more relevant for this compound, suggesting that an increase in the interaction strength could arise from the insertion of the hydroxyl group at the end of the alkyl chain.

Regarding **(3)**, we observed that increasing the alkyl chain length enhances interaction with the minor groove and mitigates cooperative binding. The competitive binding assay with groove inhibitors supports this information. A similar pattern of hydrogen-bond contributions to **(2)** could be observed due to structural similarity. In comparison with **(2)**, **(3)** can interact similarly with more restrictions of cooperative binding due to steric hindrance from the long alkyl chain. AT-rich regions in the minor groove are drastically preferred over GC-rich regions (GS 58.44 for AT-rich and 29.58 for GC-rich). In the major groove, interactions are possible with both AT and GC-rich regions indistinctively (GS 49.74 for AT-rich and 45.67 for GC-rich), but to a lesser extent compared to AT-Rich in the minor groove.

### DNA cleavage assays

An optimization of the cleavage conditions for all compounds was performed using a univariate analysis mode, in which one condition was varied at a time. Parameters such as time, concentration, temperature and ionic strength were evaluated due to their major influence on catalytic activity (Joyner et al. 2011). In contrast, the other variables were kept constant across experiments (Figures S15 and S16). Initially, the effect of the sodium L-ascorbate concentration on the compounds’ cleavage activity was evaluated (Figure S15A). All three coordination compounds required 50 µmol L^−1^ of ascorbate to achieve maximum cleavage activity. These results suggest that the copper(II) coordination sphere remains intact under the assay conditions, as confirmed by stability tests. This stability limits the metal center’s ability to induce DNA damage through hydrolytic mechanisms. Furthermore, the data indicate that these compounds can cleave DNA primarily through oxidative mechanisms, involving the generation of reactive oxygen species (ROS) via a Haber–Weiss-like cycle in the presence of a reducing agent (Prosser et al. 2021).

The effect of pH on DNA cleavage was also evaluated (Figure S15B). All compounds were capable of cleaving DNA within the range of 6 to 9. However, pH 9 proved too high for compound **2**. Compound **1** exhibited greater activity at pH 6, whereas the other complexes were more effective at pH 8, so pH 8 was chosen for subsequent cleavage experiments to allow better comparison between them.

The effects of concentration and temperature were also evaluated for all three compounds (Figures S15C and S15D). As expected, experiments performed at 37 °C were more effective than those at 25 °C, as higher temperature enhances degradation kinetics, resulting in higher cleavage levels at body temperature. Regarding compound concentration, compound **2** was more effective than **1**, which showed a similar concentration-dependent behavior to **3**. These results suggest that the presence of the alkyl chain improves DNA cleavage activity by facilitating different modes of interaction with the DNA structure. However, compound **3,** with a longer alkyl chain, may exhibit poorer interaction due to solubility issues and steric hindrance effects.

The effect of time was evaluated to determine a kinetic constant for DNA degradation by the metal complexes (Figure S16A). Compound **2** exhibited the highest kinetic constant (*k*_obs_ = 0.88 ± 0.09 h^−1^), followed by **1** (*k*_obs_ = 0.54 ± 0.06 h^−1^) and **3** (*k*_obs_ = 0.50 ± 0.03 h^−1^). All compounds showed an exponential decay of supercoiled DNA (FI) and a corresponding increase in open circular DNA (FII). Massoud et al. (2013) developed open-sphere copper(II) compounds containing pyridines that showed K_obs_ values of the same order of magnitude. Notably, there is almost no variation between one and two hours of reaction, so this time interval was chosen for the subsequent DNA interaction experiments.

The optimized cleavage conditions were used to test the efficacy of the compounds (Fig. 4A—for the same conditions without sodium L-ascorbate, check Figure S16B). Under these conditions, compounds **2** and **3** achieved maximum DNA cleavage at a concentration of 20 µmol L^−1^, while compound **1** required 50 µmol L^−1^ to reach maximum activity. For **3**, the compound-DNA complex precipitated at concentrations above 30 µmol L^−1^ due to the presence of the long alkyl chain. Under identical experimental conditions, without the addition of sodium L-ascorbate, no activity was observed.

The effect of ionic strength on DNA cleavage was also assessed (Fig. 4B). This experiment is vital because cationic species could inhibit DNA cleavage by the compounds, as cations would interact with the negatively charged phosphate backbone of the DNA, thereby repelling the compounds through Coulombic repulsion. All compounds were directly affected by ionic strength, with higher salt concentrations leading to reduced DNA cleavage. These results suggest that electrostatic interactions are essential for the compounds’ proximity to the DNA structure, facilitating ROS generation.

The effect of ROS scavenger species was also tested to identify which reactive oxygen species are generated by the Haber–Weiss-like cycle (Fig. 4C). With the presence of DMSO, a known scavenger of hydroxyl radical (·OH), the activity of compounds **1** and **2** was reduced, while that of **3** was only partially inhibited. Regarding mannitol, a more pronounced inhibition was observed for compound **3**. This suggests that **3** may be more prone to autocleavage, leading to the formation of peroxyl radicals (ROO·) from the long alkyl chain. Such autocleavage was expected, as the long alkyl chain would be less stable than the other compounds. In addition, Cu^II^/Cu^I^ reduction is more favorable for **3** than for the different compounds, leading to uncontrolled cleavage. Histidine inhibited the activity of all compounds, suggesting that singlet oxygen (^1^O_2_) is the main reactive species generated during the cycle, as observed with other copper(II) complexes (Chen et al. 2011; Zhou et al. 2016). Meanwhile, the presence of superoxide dismutase one did not cause a statistically significant difference from the positive control, suggesting that the superoxide anion (·O_2_^−^) does not participate in the reaction.

A DNA groove-interaction study of these compounds was also carried out using a competitive assay with classical groove binders (Fig. 4D). 4′,6-diamidino-2-phenylindole (DAPI) was used for minor groove, and methyl green (MG) for significant groove interactions (Zhou et al. 2016). The competitive assay showed that **1** can interact with both grooves, but as the alkyl chain size increases, the compound preferentially interacts with the minor one. Notably, compound **3** is unaffected by the presence of the primary groove binder, suggesting that it specifically interacts with the minor groove region.

## Conclusions

We successfully reported two unprecedented copper(II) complexes (**2** and **3**) synthesized using two long-chain ligands, **L**^**C6OH**^ and **L**^**C10OH**^. Both ligands derive from **bpma** (bis(pyridylmethyl)amine), forming the same coordination environment as in the reported complex **1**, which was used as the reference structure for analysis. The ligands and complexes were fully characterized using appropriate physicochemical techniques. Spectrophotometric studies of the interaction between salmon sperm DNA and the complexes (K_b_: **1** = 4.24 × 10^4^ L mol^−1^, **2** = 1.92 × 10^4^ L mol^−1^, and **3** = 1.15 × 10^4^ L mol^−1^) suggest a groove interaction between the genetic material and **1**,** 2**, and **3**. This affirmation is supported not only by similar examples in the literature but also by in silico analyses and tests using groove binders such as methyl green and DAPI. Moreover, compounds **1**,** 2**, and** 3** exhibited DNA-cleavage activity, with compound **2** showing the highest activity. These results indicate that the presence of an alkyl chain enhances the targeting of the molecule onto the DNA to an extent in which the chain length is not long enough to generate solubility issues. With the appropriate alkyl chain length, more hydrophobic interactions with the DNA structure are possible, increasing interaction strength and providing more binding modes. In general, these findings support the development of well-designed compounds as antitumoral agents in future studies.

## Supplementary Information

Below is the link to the electronic supplementary material.

Supplementary file1 (DOCX 7965 KB)

## Data Availability

No datasets were generated or analysed during the current study.

## Abbreviations

bpma: Bis(2-pyridilmethyl)amine
CD: Circular dichroism
DAPI: 4′,6-Diamidino-2-phenylindole
DMSO: Dimethyl sulfoxide
DNA: Desoxyribonucleic acid: EDTA: ethlyenediaminetetraacetic acid
L^C10OH^: 10-(Bis(pyridyl-2-methyl)amino)decan-1-ol
L^C6OH^: 6-(Bis(pyridyl-2-methyl)amino)hexan-1-ol
MG: Methyl green
ROS: Reactive oxygen species
SOD: Superoxide dismutase 1
TBE: TRIS–borate-EDTA
TRIS: Tris(hydroxymethyl)aminomethane hydrochloride
