# Supplementary material for: Fine-tuning alkyl chain length in copper(II) complexes: effects on DNA binding and catalytic cleavage
Source: Biometals. 2026 Jun 18;39(5):1903–19. doi: 10.1007/s10534-026-00840-8 (PMC13518454; doi:10.1007/s10534-026-00840-8)
Supplement: Supplementary file 1 [file 10534_2026_840_MOESM1_ESM.docx]

Supporting Information for

**Fine-Tuning Alkyl Chain Length in Copper(II) Complexes: Effects on DNA Binding and Catalytic Cleavage**

ss

Maiara I. N. dos Santos^a,#^, Matheus S. S. Paqui^b,#^, Giliandro Farias^d^, Edinara Luiz^a,e^, Ronny R. Ribeiro^f^, Hernan Terenzi^b,c*^, Fernando R. Xavier^a,*^

*^a^Chemistry Department, Universidade do Estado de Santa Catarina (Udesc), Joinville – SC, Brazil.*

*^b^Chemistry Department, Universidade Federal de Santa Catarina (UFSC), Florianópolis – SC, Brazil.*

*^c^Biochemistry Department, CCB, Universidade Federal de Santa Catarina (UFSC), Florianópolis – SC, Brazil.*

*^d^Department of Materials Physics and Mechanics, Universidade de São Paulo (USP), São Paulo – SP, Brazil.*

*^e^Department of Chemistry, KU Leuven, Celestijnenlaan 200F, Leuven, 3001, Belgium.*

*^f^Chemistry Department, Universidade Federal do Paraná (UFPR), Curitiba – PR, Brazil.*

^#^Both authors contributed equally to this work.

*Corresponding author^1^: [fernando.xavier@udesc.br](mailto:fernando.xavier@udesc.br); phone number: +55 47 3481-7654

*Corresponding author^2^: [hernan.terenzi@ufsc.br](mailto:hernan.terenzi@ufsc.br); phone number: +55 48 3721-6423

_____________________________________________________

**Table of Contents**

1	Ligand synthesis and Characterization	2

1.1	Ligand synthesis	2

1.2	Infrared Spectroscopy (IR)	5

1.3	Nuclear Magnetic Resonance	6

2	Complex characterizations	12

2.1	Infrared Spectroscopy	12

2.2	TD-DFT calculations	14

2.3	Electronic and Electrochemical data	15

3	DNA Assays	21

_____________________________________________________

# 1 Ligand synthesis and Characterization

## 1.1 Ligand synthesis

**1.1.1 Synthesis of** **bis(2-pyridilmethyl)amine (bpma)**

The compound was synthesized following the method proposed in the literature (Horn Junior 2000). In a round-bottomed flask containing 40 mL of methanol on an ice bath, 4.73 mL (50 mmol; 107.11 g mol^-1^) of 2-carboxypyridylaldehyde and 5.20 mL (50 mmol; 108.14 g mol^-1^) of 2-aminomethylpyridine, previously distilled at reduced pressure, were added. The reaction was left to stir for approximately 1 hour, and then 1.891 g (50 mmol; 37.83 g.mol-1) of sodium borohydride was slowly added, and the stirring was continued for 18 hours at room temperature. Concentrated HCl was then added until the pH reached 4, and a white precipitate was observed. The solution was concentrated in a rotary evaporator at approximately 50 °C. After removing most of the solvent, distilled water was added to solubilize the products formed. This solution was subjected to successive extractions with dichloromethane, and in the first extractions, the organic phase became colorless and was discarded. The aqueous phase was then placed in a 250 mL flask under agitation, and sodium carbonate was added until the pH reached 10. A new extraction was carried out with dichloromethane, followed by the addition of anhydrous MgSO_4_, and the solution was left to stand for 30 minutes. The organic phase was filtered and concentrated in a rotary evaporator at 50 ºC to obtain a yellowish oil, which was placed under vacuum to remove traces of solvent. Yield 60% (30 mmol; 199.25 g mol^-1^).

**1.1.2 Synthesis of** **6-(bis(pyridyl-2-methyl)amino)hexan-1-ol (L^C6OH^)**

The compound in question, **L^C6OH^**, was obtained based on the literature (Melotti et al. 2021). In a round-bottomed flask with 100 mL, 30 mL of acetonitrile were added 1.477 g (8.16 mmol; 181.07 g mol^-1^) of 6-bromo-hexan-1-ol (yellow color), 1.382 g (6.94 mmol; 199.25 g mol^-1^) of **bpma** (intense yellow color) and 2.255 g (16.32 mmol; 138.21 g mol^-1^) of potassium carbonate. After adding all reagents, the solution was stirred under reflux at 85 °C for 24 hours. After this time, the reactions were allowed to cool to room temperature, and the initial yellow solution turned into a light brown color. The solvent was evaporated under reduced pressure and washed with ethyl acetate and saturated NaHCO_3_. The organic phase was dried over anhydrous MgSO_4_, filtered and the solvent removed under reduced pressure; a small aliquot was removed for analysis via thin layer chromatography, (TLC). The analysis showed the presence of by-products separated by chromatography. The reaction products were added to the top of a glass column with an internal diameter of 03 cm and containing 11 cm of 200 mesh flash silica, using ethyl acetate (300 mL) as the first eluent to remove the by-product, followed by 100 mL of methanol. The interest product was dried using a high vacuum system, resulting in a light brown oil. Yield 51% (3.5 mmol; 299.41 g mol^-1^).

**1.1.3 Synthesis of** **10-(bis(pyridyl-2-methyl)amino)decan-1-ol (L^C10OH^)**

In a 100 mL round-bottom flask, 1.186 g (5.0 mmol; 237.18 g mol^-1^) of 10-bromodecan-1-ol, 1.737 g of potassium carbonate (12.5 mmol; 138.21 g mol^-1^), and 1.036 g of **bpma** (5.0 mmol; 199.25 g mol^-1^), were mixed in 50 mL of acetonitrile and refluxed under magnetic stirring for 36 hours. The solvent was removed under reduced pressure, and the mixture was transferred to a separating funnel, then extracted three times with 40 mL of aqueous HCl (2.0 mol L^-1^). The organic layer was discarded, and the pH of the aqueous layer was adjusted to 10 with solid potassium carbonate, at which point two phases were observed. The mixture was washed three times with 30 mL portions of ethyl acetate. The organic layers were combined, dried over anhydrous MgSO_4_, and the solvent was removed under reduced pressure, yielding a brownish oil that was used without further purification. Yield: 85 % (1.008 g, 4.25 mmol; 355.52 g mol^-1^).

## 1.2 Infrared Spectroscopy (IR)

Several bands are commonly present in the IR spectra of **bpma**, **L^C6OH^**, and **L^C10OH^** (Figure S1 and Table S1). Stretches attributed to the pyridine rings, such as C-H, C=N, and C=C, are visible at ~3110 cm^-1^ and ~1590 to 1430 cm^-1^, respectively. Likewise, the band at 1130 cm^-1^, attributed to N-H stretch, is present in all three spectra, while the band at 3300 cm^-1^ in the **bpma** spectrum is replaced by a broad O-H stretching band in the same region in **L^C6OH^** and **L^C10OH^**. These main differences corroborate the incorporation of the aliphatic alcohol in the amine group.

**Figure S1** Infrared Spectra of Ligands: **bpma** (black), **L^C6OH^** (blue); **L^C10OH^** (red).

**Table S1** Attributions of IR bands and frequencies for **bpma**, **L^C6OH^,** and **L^C10OH^.**

| **Wavenumber / cm^-1^** | | | |
| --- | --- | --- | --- |
| **Attribution** | **bpma** | **L^C6OH^** | **L^C10OH^** |
| *ν* (O-H) | - | 3330 | 3322 |
| *ν* (N-H) | 3300 | - | - |
| *ν* (C-H_ar_) | 3067-3015 | 3061-3013 | 3058-3018 |
| *ν* (C-H) _aliph_ | 2915-2830 | 2930-2857 | 2980-2762 |
| *ν* (C=N e C=C) | 1592-1434 | 1593-1431 | 1592-1434 |
| *δ* (CH_2_) | - | - | - |
| *ν* (C-N) | 1134 | 1128 | 1134 |
| *ν* (C-O) | - | 1049 | 1050 |
| *δ* (C-H_ar_) | 764 / 630 | 760 / 620 | 768 / 615 |

## 1.3 Nuclear Magnetic Resonance

All ligands were characterized by ^1^H NMR. The doublet at 8.55 ppm (a) in the **bpma** spectra (Figure S2) is assigned to the hydrogens bonded to the α-carbon in the pyridine groups. The signal labeled as “b” corresponds to the hydrogens at *para-*position relative to the pyridine nitrogen. At the same time, the doublet at “c” is consistent with vicinal hydrogens of the non-hydrogenated carbon. Therefore, the signal at 7.17 ppm should correspond to the carbon at the *meta-*position. The singlet at 3.99 ppm agrees with methylene carbons between pyridine and amine groups, while the signal at 2.70 is most likely attributed to the amine protons.

**Figure S2** ^1^H NMR of **bpma** (400 MHz, CDCl_3_) δ (ppm): 8.55 (d, 2H, *J* = 4.61 Hz); 7.65 (t, 2H, *J* = 8.00 Hz); 7.36 (d, 2H, *J* = 8.00 Hz); 7.17 (t, 2H, *J* = 6.26 Hz); 3.99 (s, 4H); 2.70 (s, 1H).

Both **L^C6OH^** and **L^C10OH^** ^1^H NMR spectra (Figures S3 and S4) show signals at higher frequencies, consistent with aromatic hydrogens. These are similar to those previously described for **bpma** and follow the same assignments as presented in Figure S2 – signals “a”, “b”, “c”, and “d”. The methylene hydrogens between the amine and pyridine groups appear at 3.79 ppm (e). The triplet “f” is related to protons α to the hydroxyl group and is observed at a higher chemical shift due to the electronegativity of oxygen. Similarly, “g” refers to hydrogens α to the amine group. The remaining methylene hydrogens of the carbon chain are labeled as “i” and “j”. Finally, the singlet at “h” is assigned to the alcohol hydrogen.

**Figure S3** ^1^H NMR of **L^C6OH^** (400 MHz, CDCl_3_) δ (ppm): 8.49 (d, 2H, J= 4.8 Hz); 7.64 (t, 2H, J= 7.6Hz); 7.52 (d, 2H, J= 7.7 Hz); 7.13 (t, 2H, J= 6.42 Hz); 3.79 (s, 4H); 3.58 (t, 2H, J= 6.6 Hz); 2.52 (t, 2H, J = 7.28 Hz); 2.46 (s, 1H); 1.55 – 1.48 (m, 4H); 1.30 – 1.27 (m, 4H).

The **L^C10OH^** ^1^H NMR spectrum (Figure S3) differs mainly from **L^C6OH^** by the higher integration value of the signals labeled as “j” in Figure S2. This multiplet at 1.27 ppm, integrating for eight additional hydrogens, corresponds to the extended carbon chain in **L^C10OH^** relative to **L^C6OH^**. As the previous spectra have been thoroughly discussed above and this ligand has already been fully characterized, we did not provide a peak-by-peak assignment for **L^C10OH^**. However, its multiplicity, chemical shift, and integration values are in good agreement with the literature.

**Figure S4** ^1^H NMR of **L^C10OH^** (400 MHz, CDCl_3_) δ (ppm): 8.51 (d, 2H, *J*= 4.69 Hz); 7.64 (t, 2H, *J*= 7.85 Hz); 7.54 (d, 2H, *J*= 8.14 Hz); 7.13 (d, 2H, *J*= 6.42 Hz); 3.80 (s, 4H); 3.62 (t, 2H, *J*= 6.50 Hz); 2.52 (t, 2H, *J*= 7.28 Hz); 2.26 (s, 1H); 1.58-1.50 (m, 4H); 1.30-1.27 (m, 13H).

^13^C NMR and DEPT-135 analysis were performed for **bpma**, **L^C6OH^** and **L^C10OH^**. In all cases, the signal at ~159 ppm (Figures S5-S7) corresponds to carbon atoms without attached hydrogens in the pyridine groups. Its absence in the DEPT analysis confirms this. The signs at 149.3 (2), 121.9 (5), and 136.4 (3) ppm in the bpma spectra correspond to the *ortho*-, *meta*-, and *para*-positions relative to the pyridine nitrogen. Also, the methylene carbon in **bpma** labeled as “6” appears at 54.6 ppm, in agreement with the peak observed at the negative phase of the DEPT-135 spectrum (Figure S5).

**Figure S5** ^13^C NMR (top) and DEPT-135(bottom) (100 MHz, CDCl_3_) of **bpma** δ (ppm): 159.4; 149.3; 136.4; 122.3; 121.9; 54.6.

Regarding the ^13^C NMR spectra of **L^C6OH^** and **L^C10OH^**, the signals described above are also present (Figure S6 and Figure S7). The sign at ~62 ppm is assigned to the CH_2_ group between the pyridine and amine groups, while the remaining signals correspond to the 6- or 10-carbon chain in **L^C6OH^** and **L^C10OH^**, respectively. Once again, we emphasize that all chemical shifts and signal counts are in good agreement with previously reported data for these ligands.

**Figure S6** ^13^C NMR (top) and DEPT-135 (bottom) (100 MHz, CDCl_3_) of **L^C6OH^**

δ (ppm): 159.9; 148.8; 136.4; 122.9; 121.9; 62.6; 60.4; 54.3; 32.6; 26.9; 26.9; 25.4.

**Figure S7** ^13^C NMR (top) and DEPT-135 (bottom) (100 MHz, CDCl_3_) of **L^C10OH^** δ (ppm): 160.1; 148.8; 136.3; 122.8; 121.8; 62.8; 60.4; 54.5; 32.8; 29.4; 29.4; 29.3; 29.3; 27.2; 27.0; 25.7.

# 2 Complex characterization

## 2.1 Infrared Spectroscopy

**Figure S8** IR spectra simulated by DFT (orange) and experimental for **1** (black), **2** (blue), and **3** (red).

| **Wavenumber (cm^-1^)** | | | | | | |
| --- | --- | --- | --- | --- | --- | --- |
| Attribution | **1 Exp.** | **1 Calc.** | **2 Exp.** | **2 Calc.** | **3 Exp.** | **3 Calc.** |
| ν (N-H) | 3240 | 3480 -3446 | - | - | - | - |
| ν (O-H) | - | - | 3523 | 3673-3670 | 3525 | 3668-3664 |
| ν (C-H_ar_ e C-H_aliph_) | 3115-2853 | 3151-2941 | 3055-2862 | 3150-2889 | 3081-2854 | 3149-2885 |
| ν (C=N e C=C) | 1609-1444 | 1611-1430 | 1611-1447 | 1654-1442 | 1608-1445 | 1611-1430 |
| (CH_2_) | - | - | 1385-1150 | 1422-1154 | 1394-1161 | 1427-1153 |
| ν (Cl-O) | 1050 | - | 1075 | - | 1078 | - |
| δ (C-H_ar_) | 768 - 620 | 1006 - 754 | 764 - 621 | 892 - 745 | 759 / 621 | 892 - 752 |

**Table S2** Attributions of IR bands and frequencies for **1**, **2** and **3** (experimental and calculated).

## 2.2 DFT and TD-DFT calculations

**Scheme S1** Molecular arrangement of the donor atoms surrounding the copper. Blue bonds are the axial position (longer length) for **1**, **2**, and **3**. (a) represents the upper left ligand, and (b) the bottom right ligand.

| **Bond length / Å** | | | |
| --- | --- | --- | --- |
|  | **1** | **2** | **3** |
| Cu–N_amine(a)_ | 2,107 | 2,365 | 2,226 |
| Cu–N_amine(b)_ | 2,372 | 2,202 | 2,367 |
| Cu–N_py1(a)_ | 2,482 | 2,139 | 2,513 |
| Cu–N_py2(a)_ | 2,019 | 2,019 | 2,012 |
| Cu–N_py1(b)_ | 2,044 | 2,496 | 2,154 |
| Cu–N_py2(b)_ | 2,026 | 2,016 | 2,019 |

**Table S3** Selected bond length (Å) calculated for **1**-**3**. (a) and (b) are related to each unit of ligand on **1-3**. (a) is superior left and (b) inferior right. Py_1_ is *trans* to py_2_.

## 2.3 Electronic and Electrochemical data

**Figure S9** Electronic Spectra of **1**, **2** and **3** and their respectively ligands in CH_3_CN. Conditions: [**1** and **2**] = 2.5×10^-5^ mol L^-1^, [**bpma** and **L^C6OH^**] = 1.5×10^-4^ mol L^-1^; [**3** and **L^C10OH^**] = 5.0×10^-5^ mol L^-1^.

|  | **Electronic data** | | **Electrochemical parameters** | |
| --- | --- | --- | --- | --- |
| **Complex** | **λ_max_ (nm)** | **ε (L mol^-1^ cm^-1^)** | ***v* (mV s^-1^)** | ***E*_1/2_ (V *vs* NHE)** |
| **1^a^** | 255  280*  634 | 15732  5000  81 | 25 | -0.12 |
| **2^b^** | 258  280*  676 | 18460  7500  142 | - | -0.06  0.07 |
| **3^b^** | 258  280*  610 | 13956  7500  75 | - | -0.19  0.07 |

**Table S4** Electronic and electrochemical data for **1-3** in CH_3_CN. *shoulder; ^a^ data collected by cyclic voltammetry; ^b^ data collected by square wave;

|  | **Energy** | |  |  |
| --- | --- | --- | --- | --- |
| **State** | **eV** | **nm** | ***f*** | **Attribution** |
| **1** | | | | |
| S_1_ | 1.09 | 1136 | 0.00002 | *d-d* |
| S_2_ | 2.19 | 566 | 0.00001 | *d-d* |
| S_20_ | 4.33 | 286 | 0.01150 | MLCT e LMCT |
| S_24_ | 4.60 | 269 | 0.00449 | LMCT e *π-π** |
| S_32_ | 4.75 | 261 | 0.02042 | MLCT e *n-π** |
| S_47_ | 5.12 | 242 | 0.04393 | LMCT*, n-π** e *π -π** |
| S_48_ | 5.20 | 238 | 0.11841 | LMCT*, n-π** e *π -π** |
| **2** | | | | |
| S_1_ | 0.93 | 1339 | 0.00019 | *d-d* |
| S_2_ | 2.02 | 612 | 0.00024 | *d-d* |
| S_3_ | 2.03 | 608 | 0.00015 | *d-d* |
| S_4_ | 2.17 | 571 | 0.00005 | *d-d* |
| S_5_ | 3.43 | 362 | 0.00627 | LMCT *e d -d* |
| S_8_ | 3.86 | 321 | 0.01076 | *π-π** |
| S_26_ | 4.24 | 292 | 0.02228 | LMCT e *π-π** |
| **3** | | | | |
| S_1_ | 0.92 | 1348 | 0.00023 | *d-d* |
| S_2_ | 0.92 | 622 | 0.00030 | *d-d* |
| S_3_ | 2.01 | 616 | 0.00013 | *d-d* |
| S_4_ | 2.13 | 581 | 0.00007 | *d-d* |
| S_5_ | 3.43 | 408 | 0.00209 | LMCT |
| S_6_ | 3.36 | 369 | 0.00744 | LMCT e *d-d* |
| S_8_ | 3.71 | 335 | 0.08974 | LMCT |
| S_25_ | 4.14 | 299 | 0.01588 | *π-π** |

**Table S5** Data of TD-DFT/TDA excitations for complexes **1**-**3**.

**Figure S10** Experimental (CH_3_CN, 1 – black, 2 – blue, 3 – red) and theoretical (orange) absorption spectra convoluted with Gaussians of 50 nm width for **1** (top), **2** (middle), and **3** (bottom).

**Figure S11** Electronic spectra of **1**, **2**, and **3**, in CH_3_CN/HEPES buffer (50%, v/v) over two hours. Each spectrum was collected every 08 minutes. [Complex] = 1×10^4^ mol L^-1^.

**Figure S12 –** Electronic Paramagnetic Resonance spectra and parameters of **1-3** in CH_3_CN at 77 K. Experimental, black lines.

# 3 DNA Assays

**Figure S13. A** bpma spectral variation with ssDNA titration (left). [bpma] = 1.5 ×10^-4^ mol L^-1^. Benesi-Hildebrand (BH) 1/(A-A_0_) vs 1/[DNA] (right). **B** Spectral variation and BH plot for L^C6OH^ under the same conditions as **A**. **C** Benesi-Hildebrand plots at 254 nm for complexes **1-3**.

**Figure S14** Best scored docking structures for **A (1)**, **B (2)** and **C (3)** with the dodecamer **2BDNA**, considering minor and major groove sites for interaction along with the sequence.

| **(1)** | | | | | |
| --- | --- | --- | --- | --- | --- |
| **Groove** | **AT/GC** | **Sol. Number** | **GoldScore** | **Hydrogen B.** | **Van der Waals** |
| Minor | AT | 18 | 43.43 | 0.00 | 31.58 |
| Minor | GC | 3 | 37.55 | 0.00 | 27.31 |
| Major | AT | 1 | 23.63 | 1.65 | 15.99 |
| Major | GC | 4 | 24.01 | 6.00 | 13.10 |

| **(2)** | | | | | |
| --- | --- | --- | --- | --- | --- |
| **Groove** | **AT/GC** | **Sol. Number** | **GoldScore** | **Hydrogen B.** | **Van der Waals** |
| Minor | AT | 1 | 47.24 | 0.22 | 38.46 |
| Minor | GC | 1 | 53.41 | 6.00 | 40.03 |
| Major | AT | 44 | 40.47 | 8.03 | 27.84 |
| Major | GC | 8 | 40.14 | 9.72 | 26.62 |

| **(3)** | | | | | |
| --- | --- | --- | --- | --- | --- |
| **Groove** | **AT/GC** | **Sol. Number** | **GoldScore** | **Hydrogen B.** | **Van der Waals** |
| Minor | AT | 1 | 58.44 | 6.00 | 40.41 |
| Minor | GC | 12 | 29.58 | 1.35 | 26.94 |
| Major | AT | 16 | 49.74 | 5.02 | 39.74 |
| Major | GC | 29 | 45.67 | 6.00 | 32.08 |

**Table S6** Docking parameters obtained for **1**-**3** with 4 different cases: minor groove (AT-Rich), minor groove (GC-Rich), major groove (AT-Rich) and major groove (GC-Rich) (PDB 2BNA).

**Figure S15** **A** Effect of sodium L-ascorbate on supercoiled DNA cleavage by complexes **1-3.** Reaction conditions**:** DNA = 330 ng (25 µmol L^-1^), [**1-2**] = 20 µmol L^-1^, [**3**] = 10 µmol L^-1^, [Sodium L-ascorbate] = 0-50 µmol L^-1^, [Buffer] = HEPES (10 mmol L^-1^, pH 7.0), T= 37 ºC, t = 4 h. **B** Effect of pH on supercoiled DNA cleavage **1-3**. [Sodium L-ascorbate] = 50 µmol L^-1^, [Buffer] = 10 mmol L^-1^, pH 6.0 (MES), 7.0-8.0 (HEPES), 9.0 (CHES). **C** Effect of **1-3** concentration on supercoiled DNA cleavage at 25ºC. [**1-2**] = 0-100 µmol L^-1^, [**3**] = 0-50 µmol L^-1^, [Sodium L-ascorbate] = 50 µmol L^-1^, T = 25 ºC. **D** Effect of **1-3** concentration on supercoiled DNA cleavage at 37ºC. Conditions not described are the same as **A**.

**Figure S16** **A** Effect of time on supercoiled DNA cleavage by **1**-**3**. DNA = 330 ng (25 µmol L^-1^), [1-3] = 20 µmol L^-1^, [Sodium L-ascorbate] = 50 µmol L^-1^, [Buffer] = HEPES (10 mmol L^-1^, pH 7.0), T = 37 ºC, t = 0-2 h. **B** DNA cleavage by compounds **1-**3 in optimized conditions without sodium ascorbate. DNA = 330 ng (25 µmol L^-1^), [1-3] = 10-50 µmol L^-1^, [Sodium L-ascorbate] = 50 µmol L^-1^, [Buffer] = HEPES (10 mmol L^-1^, pH 8.0), T = 37 ºC, t = 2 h.

Horn Junior A. (2000) Síntese e Caracterização de Novos Complexos de Ferro com Ligantes não Simétricos N,O-Doadores como Modelos para o Sítio Ativo das Fosfatases Ácidas Púrpuras. Doctoral Thesis, 252, Federal University of Santa Catarina, Florianópolis.

Melotti M, Paqui MSS, Amorim AL. (2021) Polypyridyl iron(iii) complexes containing long alkyl chains: synthesis, characterization, DFT calculations, and biological activity. New J. Chem., *45* (29):12902–12914 <https://doi.org/10.1039/D0NJ00895H>
